# A metabolically engineered spin-labeling approach for studying glycans on cells†

**DOI:** 10.1039/d0sc03874a

**Published:** 2020-10-20

**Authors:** Mohit Jaiswal, Trang T. Tran, Qingjiang Li, Xin Yan, Mingwei Zhou, Krishnendu Kundu, Gail E. Fanucci, Zhongwu Guo

**Affiliations:** Department of Chemistry, University of Florida 214 Leigh Hall Gainesville FL 32611 USA fanucci@chem.ufl.edu zguo@chem.ufl.edu; National High Magnetic Field Laboratory, Florida State University Tallahassee Florida 32310 USA

## Abstract

Metabolic glycan engineering (MGE) coupled with nitroxide spin-labeling (SL) was utilized to investigate the heterogeneous environment of cell surface glycans in select cancer and normal cells. This approach exploited the incorporation of azides into cell surface glycans followed by a click reaction with a new nitroxide spin label. Both sialic acid and *N*-acetylglucosamine (GlcNAc) were targeted for spin labelling. Although each of these moieties experiences a diverse and heterogeneous glycan environment, their EPR spectra and hence mobility are both characterized as a linear combination of two distinct spectra where one component reflects a highly mobile or uncrowded micro-environment with the second component reflecting more restricted motion, reflective of increased crowding and packing within the glycocalyx. What differs among the spectra of the targeted glycans is the relative percentage of each component, with sialic acid moieties experiencing on average an ∼80% less crowded environment, where conversely GlcNAc/GalNAz labeled sites reported on average a ∼50% more crowded environment. These distinct environments are consistent with the organization of sugar moieties within cellular glycans where some residues occur close to the cell membrane/protein backbone (*i.e.* more restricted) and others are more terminal in the glycan (*i.e.* more mobile). Strikingly, different cell lines displayed varied relative populations of these two components, suggesting distinctive glycan packing, organization, and composition of different cells. This work demonstrates the capability of SDSL EPR to be a broadly useful tool for studying glycans on cells, and interpretation of the results provides insights for distinguishing the differences and changes in the local organization and heterogeneity of the cellular glycocalyx.

## Abstract 2

Metabolic glycan engineering (MGE) coupled with nitroxide spin-labeling (SL) was utilized to investigate the heterogeneous environment of cell surface glycans in select cancer and normal cells.

## Graphical abstract

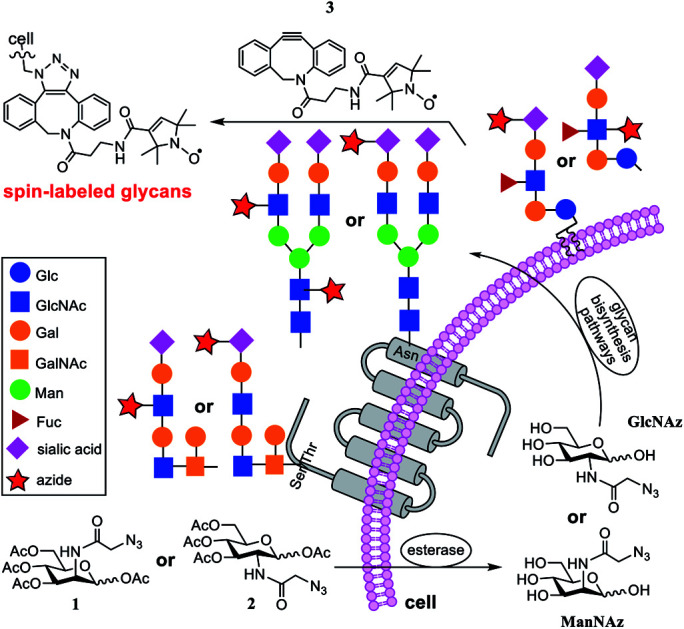

## Introduction

The cell surfaces of both eukaryotes and prokaryotes are masked by a dense layer of glycans called the glycocalyx.^1^ These glycans are attached to the outer layer of cell membranes as conjugates mainly with proteins and lipids. Being exposed at the cell surface, glycans control many important biological functions,^2^ such as signal transduction, cell adhesion, immune response, protection of hosts from pathogens, *etc.*^3–6^ Changes in the glycocalyx composition are frequently correlated with diseases. For example, thinning of the cell glycocalyx is associated with atherosclerosis prone regions;^7^ abnormal glycan expression is observed in various cancers.^8^

The varied functions of glycans are determined by their complex and diverse structures as well as the flexibility in their presentation form. As chemical receptors on the cell surface, glycans need to possess sufficient structural diversity and complexity,^9,10^ which is realized by a variety of sugar donors and glycosyltransferases or glycosidases available in nature. While each individual glycan can act as a recognition motif, it can also adopt different conformations or self-assemble to form unique patterns or signatures for molecular recognition.^11,12^ Accordingly, the spatial orientation and organization of glycans on the cell surface can dictate their bioactivity, *e.g.*, to help the immune system distinguish self from non-self.^13^ Although the structural diversity and complexity of glycans and the flexibility in their presentation forms play an important biological role, these properties significantly complicate the study of glycans.

Understanding the structures and presentation forms of glycans on the cell surface is of critical significance in glycobiology, as it can help reveal the biological functions, functional mechanisms, and structure–activity relationship of glycans, and so on. With the recent advancement of analytical technologies, *e.g.*, mass spectrometry, nuclear magnetic resonance spectroscopy and fluorescence microscopy, it has become possible to gain insights into the structure of glycans. A combination of these technologies with tools like cell surface glycan engineering,^14,15^ chemical and enzymatic glycan assembly^16,17^ and genetic engineering^18^ has been proven to be powerful for revealing the composition of the cell glycocalyx. However, defining the functional roles of spatial organization and mobility of glycans towards their biological activity has been limited.

**Fig. 1:**
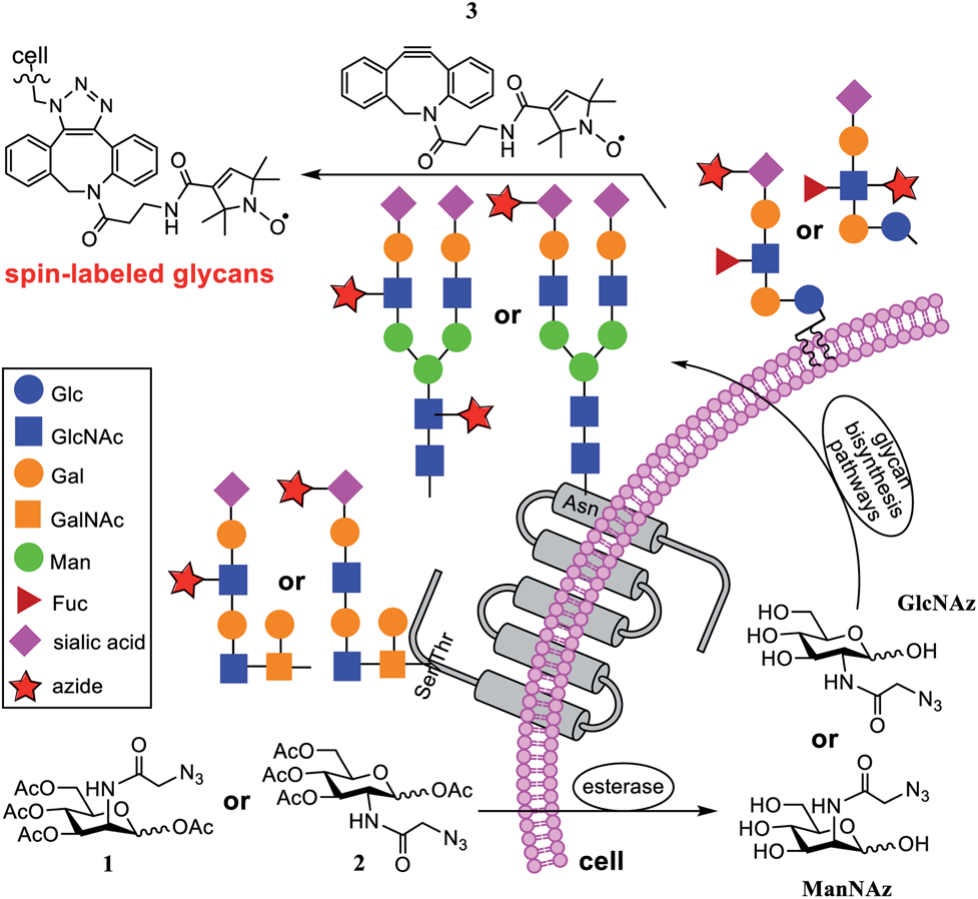
Schematic representation of MGE coupled with click-based spin labeling. Azido derivatives of *N*-acetylmannosamine **1** and *N*-acetylglucosamine **2** are taken up by cells, deacylated and converted to ManNAz and GlcNAz, respectively, and then incorporated into the biosynthetic pathways of glycans, whereby ManNAz is converted into azido-modified sialic acid (diamonds with stars) and **2** is incorporated as azido-modified GlcNAc (blue squares with red stars). The glycans on the cell surface are thereby tagged with azido groups that can selectively react with alkynes, such as **3**, under bioorthogonal conditions to introduce SLs for EPR investigations.

To address this issue, in the current research, we applied nitroxide spin-labeling coupled with electron paramagnetic resonance (EPR) spectroscopic analyses to obtain information about glycan mobility on cells. Metrics that quantify the mobility (defined as the rate of motion and order parameter) of nitroxide labels^19–26^ can be obtained from spectral line shape analyses and simulations. Here, a newly synthesized click-reactive^27–29^ nitroxide spin label (SL) was incorporated into glycans on the cell surface through metabolic glycan engineering (MGE) using azide-modified sugars as biosynthetic precursors that could be bioorthogonally coupled with the SL, followed by analyses with X-band EPR spectroscopy (see Fig. 1). This article provides a proof-of-principle study demonstrating the ability of SDSL EPR to reveal mobility differences in various glycans and cell types, suggesting that a conjugation of MGE with EPR can be a powerful tool for the investigation of glycans on cells.

## Results and discussion

### Incorporating the click-reactive nitroxide into metabolically engineered glycans

In the past three decades, MGE has emerged as a powerful tool for unraveling the mysteries of glycobiology.^27,30^ The inherent ability of MGE to chemically modify cells and then target them has facilitated its exploitation for tissue engineering, carbohydrate-based drug development, *etc.*^31–33^ Its combination with advanced analytical tools has helped gain a better understanding of the impact of structure, location, and abundance of glycans on activities.^14,15,34–37^ However, the spatial arrangements and flexibility of glycans on cell surfaces and their influence on activities remain relatively unexplored. To combine MGE labeling with EPR studies to gain insights into the organizational dynamics of glycans on cells, we selected azido sugars 3,4,5-tri-*O*-acetyl-2-*N*-azidoacetyl-2-deoxy-d-mannosyl acetate (Ac_4_ManNAz, **1**, Fig. 1) and 3,4,5-tri-*O*-acetyl-2-*N*-azidoacetyl-2-deoxy-d-glucosyl acetate (Ac_4_GlcNAz, **2**) for cell MGE and spin-labeling.

Compounds **1** and **2** are proven precursors for metabolic engineering of cells to express glycans containing unnatural *N*-azidoacetylsialic acid and *N*-azidoacetylglucosamine (GlcNAz), respectively.^38,39^ Although GlcNAc can be converted into sialic acid through the hexosamine biosynthesis pathway, it has been shown that cells cannot incorporate GlcNAc analogs carrying unnatural *N*-acyl groups, such as GlcNAz, to biosynthesize modified sialic acids because of the strict specificity of GlcNAc 2-epimerase for natural UDP-GlcNAc.^39–41^ However, GlcNAz can convert into *N*-azidoacetylgalactosamine (GalNAz) through a salvage pathway.^42^ This means that Ac_4_GlcNAz (**2**) is specific for metabolic engineering of GlcNAc/GalNAc in cell surface glycans, while Ac_4_ManNAz (**1**) leads to the exclusive labeling of azido-modified sialic acid moieties. The synthesis of **1** and **2** followed reported procedures.^43,44^

**Scheme 1:**
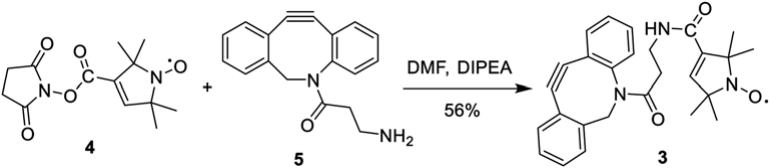
Synthesis of DBCO-SL, **3**.

Nitroxide spin-labeling was achieved *via* an orthogonal click reaction using a new functionalized SL, **3** (Scheme 1), which we call DBCO-SL. It was generated by reacting commercially available **4**, the *N*-hydroxysuccinimide (NHS) ester of *N*-oxo-2,2,5,5-tetramethylpyrroline-3-carboxylic acid (SL), with dibenzocyclooctyne-*N*-(3-oxo-propylamine) (DBCO-amine, **5**) according to a reported procedure^45^ in a good yield (56%). The product was purified by silica gel column chromatography and characterized using ^1^H NMR and HR MS data. The DBCO moiety contained a strained C/C triple bond, enabling biocompatible strain-promoted alkyne–azide cycloaddition (SPAAC) reaction with azido groups in the metabolically engineered glycans on cells.^46^ In addition, the nitroxide radical has been proved to be rather persistent,^25,47–50^ and the amide bond between SL and DBCO is typically more stable than an ester bond under various conditions.

### Approach for labelling cellular azido-glycans and EPR line shape analysis

Several cell lines were utilized for EPR investigations. The first one was a human colon adenocarcinoma cell line, Ls174T. Cell MGE and spin labeling followed the protocols established for MGE-based surface glycan labeling using other molecules.^31,37,51^ In brief, after the cells were incubated with **1** or **2** (200 μM) at 37 °C for 48 h, they were treated with **3** (DBCO-SL, 100 μM) for 1 h. Subsequently, the cells were washed with PBS buffer and then analyzed *via* X-band CW-EPR spectrometry. It was anticipated that, as shown in Fig. 1, cells would take up and process these azido sugars to eventually incorporate azido-sialic acid and azido-GlcNAc/GalNAc into various glycans and glycoconjugates.

**Fig. 2:**
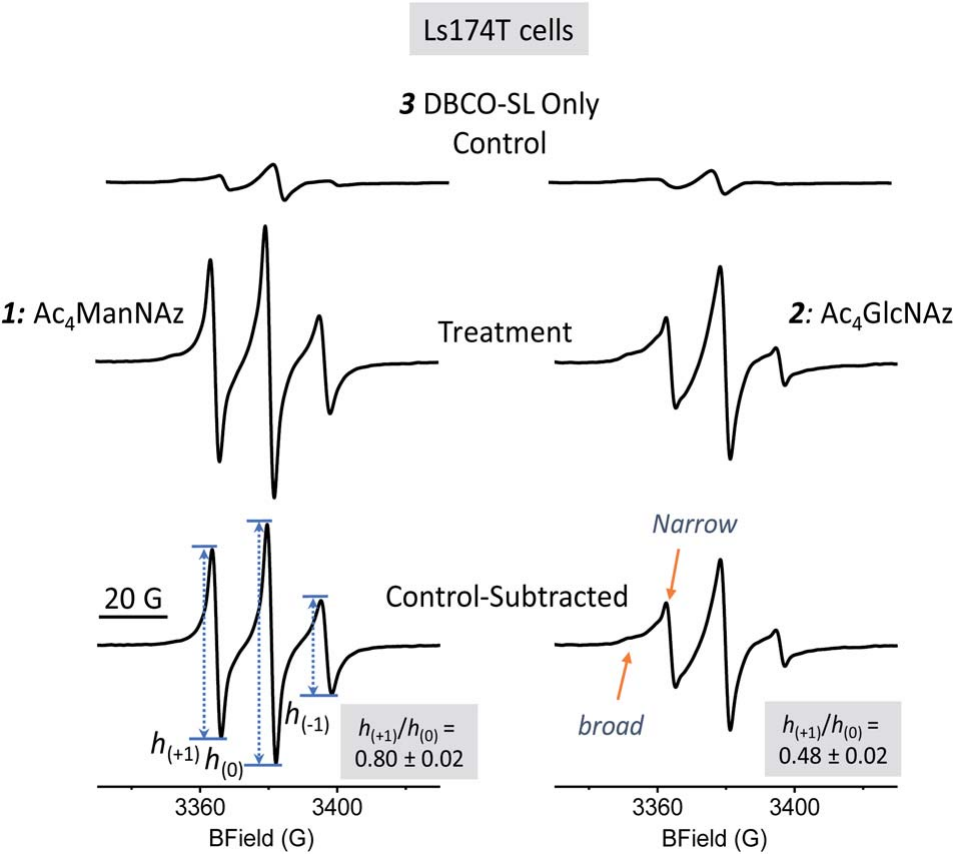
100 G X-band CW-EPR spectra of Ls174T cells treated with DBCO-SL (**3**) only (top, control), **1** and DBCO-SL (middle, left), and **2** and DBCO-SL (middle, right), and control-subtracted resultant spectra (bottom). Within a column, spectra are cell count and area-normalized to readily reflect changes in mobility, where a broader and less intense spectrum indicates lowered mobility.

As evident from Fig. 2, the EPR spectra of cells treated with an azido sugar and then DBCO-SL (treatment group) differ drastically from that of cells treated with DBCO-SL alone (the control). Moreover, the spectral shape and pattern from the treated cells are diagnostic of a nitroxide radical tethered to biomolecules experiencing restricted motion, as opposed to **3** freely tumbling in solution or a simple **3-1** or **3-2** conjugate (Fig. SI-1†), hence confirming that the detected EPR signals emerge from SL-glycoconjugates on cell surfaces. In fact, incubation of **1**-treated cells with either a sialidase or a PNGase enzyme resulted in significantly reduced EPR signal intensity of the cells, as well as appearance of labeled carbohydrates in the supernatant, which provides additional evidence for the incorporation of labeled sugars in glycans on the cell surface (Fig. SI-8†). Furthermore, the significant decrease in EPR signals of **1**-treated cells after sialidase incubation also proved that **1** was metabolically incorporated into glycans mainly (>90%) as sialic acid.

Additionally, there should be no EPR signal coming from the nitroxide if incorporated within the cell because it is well known that the reducing environment of cellular interiors destroys nitroxide radicals within minutes.^49,52–55^ Although an EPR spectrum of significant intensity was observed in the control samples (Fig. 2 top spectra), the broad feature suggests that it likely originated from partitioning of **3** nonspecifically into the cell membrane, as **3** is quite hydrophobic. To reveal the spectrum from site-specific reactions of DBCO-SL with azido-tagged sugars, control spectra were subtracted from those arising from spin-labeled MGE cells treated with **1** or **2** (Fig. 2, bottom row, herein referred to as control-subtracted spectra). These results further confirmed that SLs were incorporated into the surface glycans in live cells to enable EPR studies.

Because non-specific labeling/partitioning occurred for **3** with control cell samples not treated with **1** or **2**, we devised a sample preparation scheme for EPR data collections that allowed for reproducible and quantitative measurement of the EPR signals arising from cellular samples where the cell count was known so that we could appropriately scale the spectra (*i.e.* normalize them to the cell count) and subtract the background (control spectrum) from the treatment spectrum. Therefore, cells were counted and resuspended in PBS buffer containing 0.8% agarose for loading into capillary tubes. X-band absorption EPR spectra were collected for both treated and untreated cells using the same data collection parameters, baseline corrected using LabView software and area normalized to the cell number. For each sample, separate control spectra were collected for a given batch of cells to account for variations in cell diversity. Hence, cell count-normalized EPR spectra of cells treated with DBCO-SL only (without **1** and **2**) served as the control spectra for each cell line and each replicate sample studied within. All spectral simulations and analyses of mobility parameters refer to the appropriate control-subtracted spectra where both the treated spectra and control spectra (Fig. SI-2†) are normalized to the appropriate cell count of that sample.

### Spin label mobility varies with glycan type in Ls174T cells

Information about molecular mobility of nitroxides can be obtained by X-band EPR line shape analyses to extract empirical mobility parameters, such as the central linewidths, second moments, line intensities, *etc.*, as well as by simulations to obtain correlation time (*τ*_c_) and order parameter (*S*) values.^56–61^ In general, the CW EPR line shapes of nitroxides obtained at X-band frequency (∼9.5 GHz) are described by the four following motional regimes, fast-limit, intermediate motion, slow motion, and frozen-limit, based upon how the rate and amplitude of motion average the *g*-tensor as well as the ^14^N hyperfine coupling tensor.^56,59^ In particular, the fast-limit motional regime is typically described by correlation times <2 ns and nearly zero order parameters, resulting in “isotropic-like” spectra (Fig. SI-3†). For spectra in this fast-limit regime, the mobility line shape parameters, low-field peak to center-field peak ratio *h*_(+1)_/*h*_(0)_ or high-field peak to center-field peak ratio *h*_(−1)_/*h*_(0)_ (transition labels are shown in the bottom spectrum in Fig. 2 and SI-3†), can be used to compare the mobility among spin-labeled sites.^62–68^ Line shape parameters such as the intensities and widths of each of the three transitions can also be utilized to determine an effective correlation time, as was shown for earlier methods where sialic acid residues were selectively modified with a nitroxide label.^69^

For a nitroxide such as **3** dissolved in solvent (Fig. SI-1†), fast isotropic motion in solution generates a spectrum with three sharp lines of near equal intensity, with *h*_(+1)_/*h*_(0)_ and *h*_(−1)_/*h*_(0)_ values near 1. As motional rates slow down or become restricted in space, *e.g.*, due to bonding to a biological molecule, the EPR spectra will broaden and have diminished intensities of *h*_(+1)_ and *h*_(−1)_ transitions compared to the central *h*_(0)_ transition, resulting in lower values for these mobility parameters.^70^ Hence, for the attachment of **3** to azido-modified glycans that display fast-limit isotropic-like motion, the *h*_(+1)_/*h*_(0)_ and *h*_(−1)_/*h*_(0)_ values are predicted based upon simulation to be in the range of ∼0.6 to 1 (Fig. SI-3†). For motions of SLs that are more restricted, due to interactions with other biomolecules or restriction in the backbone mobility of the carbohydrate, intermediate motion spectra are expected. Intermediate motion spectra reveal broadened features in the low- and high-field transitions and are typically not described well by *h*_(+1)_/*h*_(0)_ and *h*_(−1)_/*h*_(0)_ values, but rather parameters such as the second moment 〈*H*^2^〉 and central linewidth Δ*H*_o_.^71^

The EPR spectra for SLs attached to **1**- and **2**-treated Ls174T cells reflect different average mobilities, with *h*_(+1)_/*h*_(0)_ values of 0.80 ± 0.02 and 0.48 ± 0.02, respectively (Fig. 2, bottom row), indicating that SLs on sialic acid moieties display on average fast-limit isotropic-like motion, while SLs attached to GlcNAc/GalNAc moieties exhibit on average intermediate regime motion. The difference in average mobility reflected by SL attachment to these two sugars agrees well with the locations of these moieties in glycans (Fig. 1). Sialic acid is usually the terminal sugar residue in a majority of glycoconjugates. Being exposed on the cell glycocalyx surface, sialic acid residues are anticipated to be innately flexible and mobile. In contrast, GlcNAc, as well as GalNAc, is usually located in positions inside the glycans and therefore is expected to encounter increased intermolecular interactions that can impart lessened spin-label flexibility. Unmistakably, the resultant EPR results reflect differences in the average local environments between spin-labeled sialic acid and GlcNAc/GalNAc, and hence the organization of the two sugar residues.

### Different environmental populations revealed for spin labeled sugars

The location and mobility of GlcNAc/GalNAc in cell surface glycans are rather diverse. For example, GlcNAc residues in the *N*-glycan inner core are directly linked to proteins, and some other GlcNAc/GalNAc residues are also close enough to the protein/lipid backbones or the cell membrane to be buried deep in the cell glycocalyx; thus they might be expected to exhibit restricted mobility or flexibility due to intermolecular and intramolecular interactions. On the other hand, GlcNAc residues within the *N*-glycan antenna or located relatively at the outer sphere of the cell glycocalyx should have increased mobility or flexibility (Fig. 1, blue squares). In addition, although sialic acid moieties typically occupy terminal positions in glycans, the crowding and packing of their local environment may differ, depending on the overall length or size and branching of the glycan in which they occur. As a result, each EPR spectrum is the average of different components in varied ratios. In fact, evidence for different spectral components is clearly seen in the EPR spectral line shapes of DBCO-SL/**2**-treated cells, which contain a broad and a narrow component (arrows in Fig. 2, bottom) reflective of two different environments for the SLs, where the narrow component reflects high mobility and the other arrow points to a spectral feature that reflects lower mobility/flexibility, consistent with spin-labels located closer to proteins or other membrane components.

**Fig. 3:**
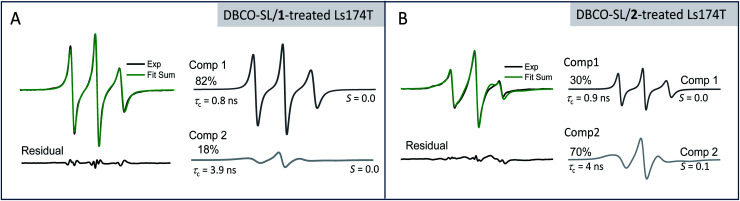
Simulation results for (A) DBCO-SL/**1**-treated and (B) DBCO-SL/**2**-treated Ls174T cells showing the experimental and simulation spectra, along with residuals and the component spectra obtained from the simulations. Although the results for order parameters approached zero, we included the ordering potential in the simulations because isotropic motion is an unreasonable expectation *a priori* given the tethering of the SL. The findings here that *S* approaches zero are likely attributed to the long tether of the SL (compound **3** when attached to the sialic acid should have many degrees of freedom around rotatable bonds) to the glycans coupled with glycan motions, which together allow for isotropic-like motion of the spin-tensor.

While the *h*_(+1)_/*h*_(0)_ mobility parameter gives insight into the *average* mobility of the SLs and spin-labeled sugars, a better understanding of the multiple component environments is obtained from spectral simulations^72^ with the *chili* and *esfit* functions utilized here. Given that the heterogeneous environment of the cell surface and the CW-spectra indicate multiple components, simulations were performed with both single and multiple component analysis to evaluate the relative populations and characterize the mobility of each population (simulation details in ESI Tables S1-1–4†). Spectral simulations for the different azido-modified sugars independently converged to results that contained two similar component spectra, but varied in the relative population of each component (Fig. 3). In each case, a fast motion component with nearly isotropic motion (*S* ∼ 0) with a correlation time of ∼0.9–0.8 ns was observed with the second spectral component having intermediate motion with *τ*_c_ ∼ 3–4 ns and *S* ∼ 0.0–0.1. Labeled sialic acid sites in Ls174T cells exhibit 82% mobile component with only 30% mobile component observed for labeled GlcNAc/GalNAc sites. Moreover, the spectral parameters for the more immobilized (*i.e.*, broad) population lacked evidence for dipolar interactions, meaning that the broad component arises from restricted mobility and not from spin–spin interactions.

### Glycan mobility varies among cells

**Fig. 4:**
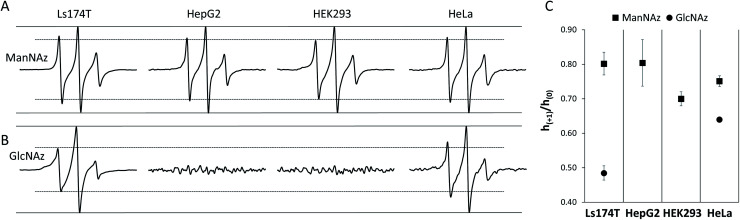
Comparison of spin label mobility for various cell and glycan types. For visual comparison, 100 G X-band EPR spectra of various cells are plotted with equally scaled central line intensities. All spectra are control-subtracted and normalized to the cell count. Data are shown for cells treated (A) with DBCO-SL/**1** or (B) with DBCO-SL/**2**. Solid lines placed at the maximum and minimum of the center-peak and dashed lines placed at the maximum and minimum of the low-field peak serve as a guide for the eye to show changes in peak height among the various cell types. (C) Mobility of spin-labeled carbohydrates in various cell lines. Higher values of the *h*_(+1)_/*h*_(0)_ mobility parameter indicate a greater degree of mobility. Error bars represent the standard deviations from 2 to 4 different batches of cells.

**Fig. 5:**
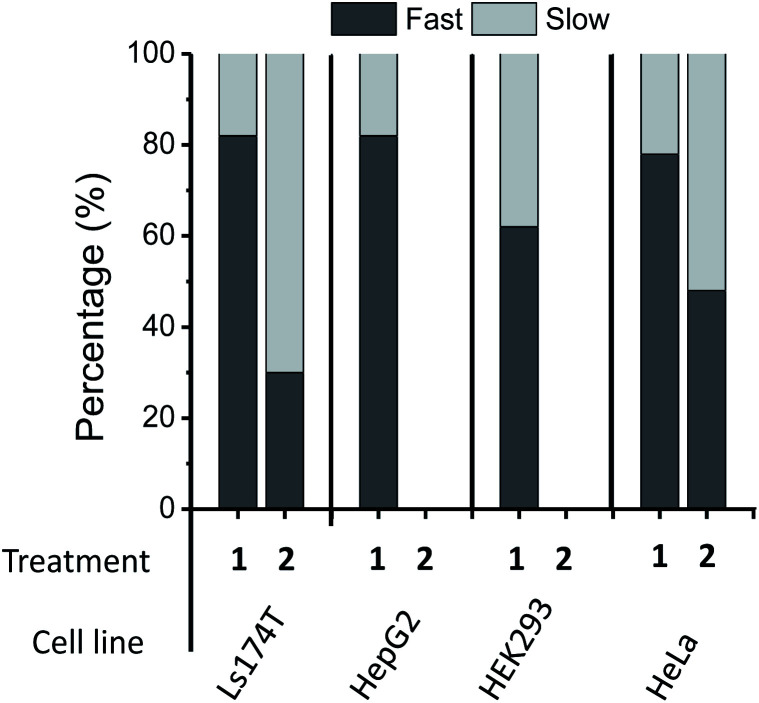
Population analysis results from two-component spectral simulations with a fast (*τ*_c_ ∼ 0.8–0.9 ns) and a slow component (*τ*_c_ ∼ 3–4 ns) for 200 μM **1**- and **2**-treated cells. Replicate samples give relative errors of ±4% for Ls174T cells, *τ* ± 6% for HepG2 cells and ±3% for HEK293 and HeLa cells. No data are shown for **2**-treated HepG2 and HEK293 cells because **2** is not a good biosynthetic precursor for MGE in these cell lines.^39,68^

In addition to LS174T cells, three other cell lines including a human hepatocyte carcinoma cell line (HepG2), a human cervical cancer cell line (HeLa) and a noncancerous human embryonic kidney cell line (HEK293) were investigated. The DBCO-SL/**1** combination was able to label all cell lines. The resulting experimental EPR spectra for the four cell lines are exhibited in Fig. 4A, and the averaged *h*_(+1)_/*h*_(0)_ mobility parameters from replicate experiments are presented in Fig. 4C. The relative populations of the mobile and restricted components are presented in Fig. 5. Remarkably, labelled sialic acid showed distinguishable average mobilities among cells, with Ls174T and HepG2 cells having similar average mobilities, with a slightly reduced mobility in HeLa cells. Noncancerous HEK293 cells exhibited a statistically significant lowest sialic acid mobility among the four cell lines probed in this study. The origin of these varied average mobilities may be attributed to the altered relative populations of the mobile and restricted components for the various cells, as determined from spectral simulations (Fig. 5). This finding suggests that the various cells have either differences in the location and packing of sialic acid moieties within the glycocalyx or that they have different metabolic engineering efficiencies, or possibly a combination of both.

Nonetheless, these observations suggest that spin-labeling EPR approaches can be reflective of differences in glycan organization and composition. These preliminary results indicate that SDSL coupled with MGE may be a useful technology to distinguish different cell types/glycosylation patterns/changes in the glycocalyx organization. As such, future experiments will be aimed at studying pairs of cell lines for cancerous and normal cells from similar tissues.

When labeling various cells with DBCO-SL and **2**, only Ls174T and HeLa cells, but not HepG2 and HEK293 cells, were efficiently spin-labeled as evidenced by their control-subtracted EPR spectra (Fig. 4B). This result is consistent with previous reports that **2** is not a good biosynthetic precursor for metabolic engineering of HEK293 and HEPG2 cells.^39,73^ Just as seen for Ls174T cells, the averaged mobility of DBCO-SL/**2**-treated HeLa cells is lower than that of DBCO-SL/**1**-treated HeLa cells (Fig. 4C). However, when comparing the spectra of DBCO-SL/**2**-treated cells, we see that HeLa cells have a markedly higher mobility than that of similarly treated Ls174T cells. Again, the differences in average mobility may arise from variations in the relative populations of the mobile and more restricted mobility components, with the mobile component comprising 30% in Ls174T cells as compared to 47% in HeLa cells (Fig. 5).

These results suggest that spin labeling of glycans with **3** is a sensitive way to probe differential packing interactions within the complex glycocalyx. Therefore, the different EPR spectra may well reflect a combination of sugar residues in two different average environments, one for the terminal sugar moieties with fewer intermolecular/intramolecular interactions and the other more crowded as reflected by the broadened spectral feature showing the contact of SLs with other biomolecules that restrict their overall motional flexibility/rate of motion.

### Probing sialic acid mobility as a function of azido sugar dose

**Fig. 6:**
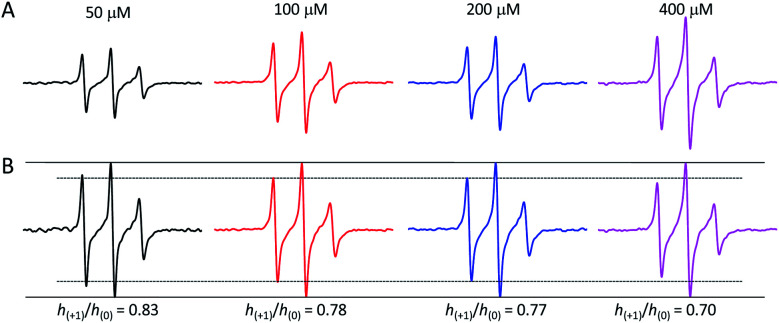
100 G X-band EPR spectra of control-subtracted Ls174T cells (A) treated with 50, 100, 200, and 400 μM concentrations of **1** and then 100 μM DBCO-SL plotted normalized to the cell count showing the increase in total spin count and (B) plotted with normalized central line intensity (solid lines) clearly showing that the *h*_(+1)_ intensity (dashed line) is decreasing with increased sugar concentration, indicating a decrease in mobility or increase in spin–spin interactions.

We also examined the influence of the concentrations of **1** on spin labeling of sialic acid on Ls174T, HepG2 and HeLa cells. Ls174T cells had resultant EPR spectra that were systematically altered as a function of sugar concentration relative to cell population, showing changes in both line shape and peak intensity (Fig. 6A and SI-5–7, and Tables SI-5–8†). For all cells, the spectral area, reflective of the total spin-count, grows with increased concentration of **1** fed to the cell culture, indicating that more azido-sugar is incorporated as sugar concentration increases (Fig. SI-5 for HepG2 and Fig. SI-6† for HeLa). By plotting spectra with normalized center-field peak intensity (Fig. 6B), we can readily see a dose-dependent decrease in low-field peak intensity for Ls174T cells. The mobility parameter *h*_(+1)_/*h*_(0)_ values of spins decrease as sugar concentration increases: 0.84 ± 0.02, 0.79 ± 0.02, 0.75 ± 0.02, and 0.70 ± 0.02 for 50, 100, 200 and 400 μM concentrations of **1**, respectively.

Spectral simulations were performed in efforts to distinguish the source of the line shape change. Both two-component and single-component fits were explored. Possible interpretations from the single component fits are as follows. In the first set of simulations, the observed reduced SL mobility upon increasing sugar concentration can be described by a single environment that has a reduced correlation time of motion resulting from increased crowding/intermolecular interactions. Secondly, simulations where the dipolar interaction is allowed to increase as a function of sugar concentration without a change in correlation time can also recapitulate the changes in the EPR line shapes, suggesting that as more azido sugars are incorporated into the glycocalyx, the line shapes broaden due to increased spin–spin interactions from nearby SLs within 20 Å (Table SI-5†). However, low temperature (100 K) X-band EPR analysis did *not* show evidence of dipolar interactions (Fig. SI-4† data), thus potentially ruling out spin–spin interactions as the source of the line shape changes.^26^

Alternatively, simulations with two spectral components (mobile fraction, *τ*_c_ ∼ 0.9 ns; intermediate mobility fraction, *τ*_c_ ∼ 4 ns) suggest that the systematic decrease in the SL mobility can arise from an increase in the relative percentage of the intermediate mobility population as azido-sugar concentration increased (Table SI-8†). The RMSD of simulations with two components is slightly improved over that of the one-component fitting. However, without further investigations at higher magnetic fields,^57,61^ we cannot rule out one explanation over the other at this time.

Nevertheless, the observed spectral changes indicate that as sugar concentration increases, a greater percentage of azido sialic acid is incorporated into the glycocalyx in the three cells studied, but only for Ls174T cells did the results imply a concomitant decrease in average mobility. Room temperature experiments of spin-labelled cells at higher frequencies such as Q-, W- or D-band could help rule out the contributing factors, and W-band experiments are planned for the future.^74,75^

## Conclusions

Site-directed spin labeling EPR technologies for studying proteins and DNAs/RNAs typically involve incorporating a reactive amino acid or nucleotide at specific sites so that EPR active labels can be attached to desired locations to gain information about the local environment reflected in measurements of label mobility, accessibility or distance between labels. However, this strategy is not readily applicable to carbohydrates as their biosynthesis is not template controlled. To achieve sugar residue-specific spin labeling of cell surface glycans for EPR investigations, we described herein a new strategy that combines MGE with bioorthogonal chemical reaction to affix SLs. Accordingly, **1** and **2** were employed for metabolic engineering of cells to express glycans carrying azido-modified sialic acid and GlcNAc/GalNAc, respectively, and SLs were then attached to the modified glycans *via* SPAAC reaction with **3**. We achieved excellent and reproducible EPR signals from labeled cells, thereby proving the feasibility of this approach. In addition, high quality data were easily obtained with a benchtop EPR spectrometer. In this study, EPR spectra were originally collected using a research grade spectrometer (Bruker E500), but the spectra presented herein were collected using a benchtop spectrometer, demonstrating the easy accessibility of this technology.

The length of the SL tether and size of the nitroxide label itself are known to impact the EPR line shape.^69^ Typically, the larger the tether length and SL size, the less the EPR spectrum reflects the motion of the backbone of a biomolecule. Given that the tether length and size in **3** are rather large, we would expect a highly mobile line shape when it is attached to a biomolecule that is not involved in significant intermolecular/intramolecular interactions, even if tethered to a surface. Hence, the broadened spectral component observed for **3** attached to sialic acid or GlcNAc/GalNAc likely arises from the restricted motion due to interaction with other molecules nearby in the glycocalyx, and as such we interpret the EPR spectra to report on the crowdedness of the local environment that can hinder motions of the SL itself along with the sugar moiety.

Furthermore, detailed analysis of the resultant EPR spectra has provided insights into the local environments and mobility of SLs to reveal new insights. For example, we found that in all probed cell lines, the average local environmental mobility of SLs incorporated with DBCO-SL and **1** was constantly higher than that of SLs incorporated with DBCO-SL and **2**, in good agreement with the different locations of sialic acid and GlcNAc/GalNAc within natural glycans. In addition, simulations of the EPR spectra of DBCO-SL/**1**- and **2**-treated cells have revealed two distinct spectral components, one mobile and the other less mobile, reflecting two average local environments for glycans that differ in their degree of crowdedness, which impacts the spin label mobility. Strikingly, the EPR spectra of LS174T and HeLa cells treated with DBCO-SL and **2** reveal different relative populations of the distribution of GlcNAc/GalNAc between the two environments. We also observed evidence in the DBCO-SL/**1**-treated cells that the HEK293 normal cells possessed lower average mobility than the cancerous cell lines investigated here, possibly indicating a less crowded environment for sialic acids on cancer cells. Taken together, these findings also support future synthetic efforts at site-specific incorporation of nitroxide labels of varying tether lengths into glycans to obtain more detailed information so as to probe more precisely the local dynamics, organization, and environment of glycans on cell surfaces.

In conclusion, MGE-based spin-labeling of cell surface glycans in conjunction with EPR spectroscopy provides a facile technology to probe glycan mobility and organization in the cell glycocalyx. Although the tether in DBCO-SL was rather long, analysis of the resultant EPR spectra provided not only insights into the degree of glycan crowding reflected by SL order parameters and rates but also organizational and spatial details of glycans reflected by the spin–spin interactions and other important information, such as a quantitative measure of the relative fractions of different environments. However, current MGE techniques using sugar derivatives such as **1** and **2** are nonselective, resulting in indiscriminative labeling of glycans and an average EPR signal of all spin-labeled glycans distributed throughout the entire cell glycocalyx. This excludes the possibility to identify specific glycans and carry out further detailed analysis of their structures. However, this issue may be addressed in the future by coupling EPR studies with mass spectrometry analysis and/or with specifically nitroxide-labeled glycans.

## Experimental

### Synthesis of *N*-[dibenzocyclooctyne-*N*-(3-oxo-propyl)]-1-oxyl-2,2,5,5-tetramethylpyrroline-3-carboxylamide (DBCO-SL, **3**)

To a solution of 1-oxyl-2,2,5,5-tetramethylpyrroline-3-carboxylate *N*-hydroxysuccinimide ester (**4**, 2 mg, 0.007 mmol) in DMF (1 mL), *N*,*N*-diisopropyl-ethylamine (DIPEA, 2.3 mg, 0.018 mmol) was added at 0 °C. After the mixture was stirred at 0 °C for 10 min, DBCO-amine (**5**, 1.6 mg, 0.006 mmol) was added, and the solution was stirred at room temperature (rt) for 2 h until **5** disappeared, as shown by thin layer chromatography (TLC). The product was purified by flash silica gel column chromatography (ethyl acetate/hexane = 1/1) to provide **3** as a colorless solid (1.5 mg, 56%). ^1^H NMR (600 MHz, CDCl_3_) *δ*: 7.72 (br, 1H), 7.44–7.34 (m, 7H), 6.43 (br, 1H), 5.19 (d, 1H, *J* = 4.3 Hz), 3.75 (d, 1H, *J* = 4.3 Hz), 3.67 (br, 2H), 2.52 (br, 1H), 2.03 (br, 1H), 1.52 (br, 6H), 0.86 (br, 6H) (note: because of the paramagnetic properties of **3**, its NMR signals were broadened; thus the integrations are relative and some of the coupling constants could not be accurately measured; Fig. SI-10†). High resolution ESI-MS (*m*/*z*): calculated for C_27_H_29_N_3_O_3_^+^ [M + H]^+^ 443.2203, found 443.2196 (Fig. SI-11†).

### Cell culture preparation

HEK293, HeLa, HepG2, and LS174T cells were cultured in Dulbecco's modified Eagle's medium (DMEM) supplemented with 100 U per mL of penicillin–streptomycin and 10% fetal bovine serum (FBS) under an atmosphere of 5% CO_2_ and 95% air at 37 °C in tissue culture flasks. After 3–4 days of culture when approximately 90% confluency was reached, the cells were peeled using Gibco™ Trypsin–EDTA (0.25%) and resuspended in a fresh culture medium. An aliquot of each cell suspension was used for cell counting with 0.4% trypan blue stain and a hemocytometer. After performing the cell counting, the required number of cells was transferred into a new culture flask for metabolic engineering experiments.

### Metabolic engineering of cells with azido sugars

Ac_4_ManAz (**1**) or Ac_4_GlcNAz (**2**) was dissolved in ethanol to form a 20 mM stock solution. An appropriate amount of each stock solution (10, 20, 40 or 80 μL) was transferred into a T-25 tissue culture flask (25 cm^2^), and the flask was put in a sterile laminar hood to allow ethanol to evaporate. Next, 4 mL of DMEM media supplemented with 100 U per mL of penicillin–streptomycin and 10% FBS was added to each flask to achieve the appropriate concentration (50, 100, 200 and 400 μM) of **1** or **2**. Thereafter, 1.25 × 10^6^ cells were added into each flask, and the flasks were incubated under the above-mentioned cell culturing conditions for 48 h. For negative control EPR samples, the cells were incubated without **1** or **2**. Subsequently, an EDTA solution (1 mM, 0.75 mL) in PBS (pH 7.4) was added to each flask to detach the cells. After 4 mL of ice-cold PBS was added to each flask, the cell suspension was transferred into a 15 mL conical tube and then subjected to centrifugation at 100*g* and 4 °C for 8 min to obtain the cell pellet. The cells were resuspended in 2% BSA in PBS (FACS buffer), counted as described above, and again centrifuged to obtain the cell pellet. Finally, the cells were resuspended in a 4% paraformaldehyde solution in PBS for 15–20 min, washed 3 times with FACS buffer, ready for the click reaction.

### Spin label click attachment to the metabolically engineered cells

DBCO-SL was dissolved in dimethyl sulfoxide (DMSO) to obtain a 1 mM stock solution, which was utilized to prepare a 100 μM DBCO-SL solution in FACS buffer. About 1 × 10^6^ cells obtained above were transferred into each 1.5 mL microfuge tube and pelleted upon centrifugation as mentioned above. The cell pellet was resuspended in 500 μL of FACS buffer containing 100 μM DBCO-SL. This mixture was kept in a shaker incubator at rt for 60 min with shaking at 300 rpm. The reaction was quenched with 1 mL of ice chilled FACS buffer. The cells were collected by centrifugation and then washed 3 times with FACS buffer (3 × 1 mL). Finally, the cells were obtained as pellets after centrifugation at 6000 rpm (2300 × *g*) for 1 min.

### Sample preparation for CW-EPR experiments

The cell pellet obtained above was resuspended in 50 μL of PBS containing 0.8% agarose. This resuspension was then immediately loaded into a 50 μL-microcapillary pipette tube (Hirschmann), and the sample was allowed to solidify before CW-EPR data collection.

### CW-EPR data collection

X-band (9.5 GHz) CW-EPR absorption spectra were collected at 30 °C using a Magnettech MiniScope MS-5000 benchtop or Bruker E500 spectrometer with a dielectric resonator. Spectra were reported as an average of 16 scans with 120 mT sweep width, 0.2 mT modulation amplitude, 100 kHz modulation frequency and 1 mW incident microwave power (2 mW incident microwave power on the Bruker E500). All of the EPR spectra were area normalized to the cell number, and all spectra were baseline-corrected and processed using the LabVIEW software provided by C. Altenbach and W. Hubbell (https://sites.google.com/site/altenbach/labview-programs). Low temperature (100 K) spectra were collected as described previously on the Bruker E500 spectrometer.^76^ High-field 240 GHz CW-EPR spectra were collected on a CW/pulsed heterodyne EPR spectrometer operated at 240 GHz at the National High Magnetic Field Laboratory (NHMFL) and are described in more detail in the ESI.†^77^

### EPR line shape simulations

EPR spectra were simulated using the *chili* and *esfit* functions of EasySpin.^72^ To determine *g*-tensor values for the new SL, **3**, low-temperature, high-field 240 GHz EPR spectra of **3** and **3-1** were obtained at the National High Magnetic Field Laboratory^77^ (Fig. SI-1E and F†) and were simulated using the *pepper* function in EasySpin. These *g*-tensor values (*g*_xx_ = 2.0084, *g*_yy_ = 2.0061, *g*_zz_ = 2.0022) were utilized as starting parameters for simulation of X-band spectra and final values differed only by 0.06%. *A*-tensor values were allowed to vary from simulations of 240 GHz spectra because **3** and **3-1** were frozen in aqueous DMSO solutions, which have different dielectric constants than aqueous solution. A set of *g*-tensor and *A*-tensor values, along with line broadening values were obtained by simulations of control-subtracted **3**/**1**-treated Ls174T spectra. Then these values for *g*- and *A*-tensor were utilized in a global fitting of all spectra such that all spectra within have a single set of *g*- and *A*-tensor with reasonable correlation times. The best fits of *A*- and *g*-tensor values were *g*_xx_ = 2.0070, *g*_yy_ = 2.0062, *g*_zz_ = 2.0033, *A*_xx_ = 6.7 G, *A*_yy_ = 6.7 G, and *A*_zz_ = 35 G. They were different from those obtained with MTSL (*g*_xx_ = 2.0089, *g*_yy_ = 2.0058, *g*_zz_ = 2.0021, *A*_xx_ = 6 G, *A*_yy_ = 6 G, *A*_zz_ = 37 G),^58–61^ a widely used nitroxide for protein labeling or with IAP (*g*_xx_ = 2.0076/2.008(4), *g*_yy_ = 2.0050/2.006(1), *g*_zz_ = 2.0023/2.002(3), *A*_xx_ = 6.2/7.5 G, *A*_yy_ = 4.6/5.9 G, *A*_zz_ = 36/35 G; two sets of values given from two different publications).^74,75^ The other parameters used in the EPR line shape simulation include linewidth (which can be reflective of spin–spin interactions), correlation time of motion (*τ*_c_), and the ordering potential C20, which was used to calculate the motional order parameter *S*.^58–61^ Simulations of the spectra were attempted first using a 1-component setup (Table SI-2†). If 1-component fit simulations could not recapitulate the features of a spectrum, 2-component simulations were utilized, which contained a fast motion component to describe the sharp peaks and a slower motion component to capture the broadened areas of the spectrum.

## Author contributions

MJ contributed to cell culturing and engineering; TTT and MZ contributed to EPR studies; KK contributed to high field EPR data collection and analysis; QL and XY contributed to compound synthesis; GEF and ZG were responsible for the overall design and supervision of this project. All authors contributed to the manuscript preparation.

## Conflicts of interest

The authors declare no competing financial interest.

## Supplementary Material

SC-011-D0SC03874A-s001
