# Supplementary material for: A metabolically engineered spin-labeling approach for studying glycans on cells
Source: Chem Sci. 2020 Oct 20;11(46):12522–32. doi: 10.1039/d0sc03874a (PMC8162880; doi:10.1039/d0sc03874a)
Supplement: SC-011-D0SC03874A-s001 [file SC-011-D0SC03874A-s001.pdf]

Electronic Supplementary Material (ESI) for Chemical Science.
This journal is © The Royal Society of Chemistry 2020

Supporting Information

for

Metabolically Engineered Spin-Labeling Approach for Studying Glycans on Cells

Mohit Jaiswal,[a†] Trang T. Tran,[a†] Qingjiang Li,[a] Xin Yan,[a] Mingwei Zhou,[a] Krishnendu Kundu,[b] Gail E. Fanucci,[a*] and Zhongwu Guo[a*]

[a] Department of Chemistry, University of Florida, 214 Leigh Hall, Gainesville, FL 32611, United States

[b] National High Magnetic Field Laboratory, Florida State University, Tallahassee, Florida 32310

[†] These authors contribute equally to the current work.

* Corresponding author e-mails: fanucci@chem.ufl.edu; zguo@chem.ufl.edu

Table of Contents

I. EPR spectra for control experiments -- SI2-5

II. EPR spectra for dose dependent experiments --- SI5-6

III. EPR spectra for labeled cells after enzyme treatment ------------------------------------- SI6-7

IV. EPR spectral simulation results --- SI7-9

V. ^{1}H-NMR and MS spctra of compound **3** -- SI10

I. EPR spectra for control experiments

- EPR spectra of free spin label 3 and 3-1 conjugate and their spin parameters

High-field continuous wave (CW) EPR spectra (Z. C. Liang, Y. Lou, J. H. Freed, L. Columbus, and W. L. Hubbell, *J. Phys. Chem. B*, 2004, 108, 17649-17659) of free SL **3** and SL-Ac_4ManNAz (**3-1**) conjugate were recorded for a ~1 mM solution of **3** in DMSO and ~1.2 mM solution of **3-1** in 1:2:1 DMSO:ethanol:PBS buffer solution on a CW/pulsed heterodyne EPR spectrometer operated at 240 GHz at the NHMFL (J. van Tol and L. C. Brunel, *Rev. Sci. Instrum.* 2005, *76*, 074101). The probe is designed to transport millimeter-wave radiation via a cylindrical HE_{11} mode corrugated waveguide to the sample, which is contained within a cylindrical glass EPR tube (sample volume 10 µl) at the field center of a 12.5 T high-resolution (10 ppm over a 10 mm sphere) superconducting magnet (Oxford Instruments). Temperature control was achieved using a continuous-flow helium cryostat and controller (Oxford Instruments). The measurements were performed at a relatively high temperature of 100 K to avoid saturation of the signal due to the long spin-lattice relaxation time at lower temperatures. Magnetic field modulation was employed at approximately 40 kHz, with a modulation amplitude of approximately 4 G. The inductive mode signal was isolated from the reflected millimeter-wave radiation using a Martin-Puplett interferometer followed by a heterodyne (phase-sensitive) detection scheme. The magnetic field strength was calibrated using the standard BDPA radical (g = 2.0037).

The EPR spectra were simulated using *pepper* functions in the program EasySpin (S. Stoll and A. Schweiger, *J. Magn. Reson.* 2006, *178*, 42-55), and the obtained spin Hamiltonian parameters are presented in Table SI-1 with simulations shown in Figure SI-1 E and F. To obtain the best agreement, magnetic field strain was employed to account for inhomogeneous broadening caused by unresolved hyperfine interactions within the relatively broad peaks. The results suggest that the g anisotropy of the SL remains largely unchanged whether it is free or attached to sugars.

Table SI-1. Simulation parameters for g-tensor and A-tensor components determined from 100 K 240 GHz spectra and from room temperature X-band spectra of **3**-labeled cells

Sample (1mM)	g_{xx}	g_{yy}	g_{zz}	A_{xx} (G)	A_{yy} (G)	A_{zz} (G)	$Hstrain_X$ (G)	$Hstrain_Y$ (G)	$Hstrain_Z$ (G)
free SL **3**	2.0084	2.0061	2.0022	5.3	4.3	36.0	21.4	7.1	3.6
**3-1** conjugate	2.0085	2.0061	2.0022	6.1	4.3	35.3	21.4	7.1	3.6
X-band simulations of **3**-labeled cells	2.0070	2.0062	2.0033	6.7	6.7	35	na	na	na
Percent error*	0.07	0.005	0.05	na	na	na	na	na	na

*Error from best X-band fitting prior to obtaining high-field parameters

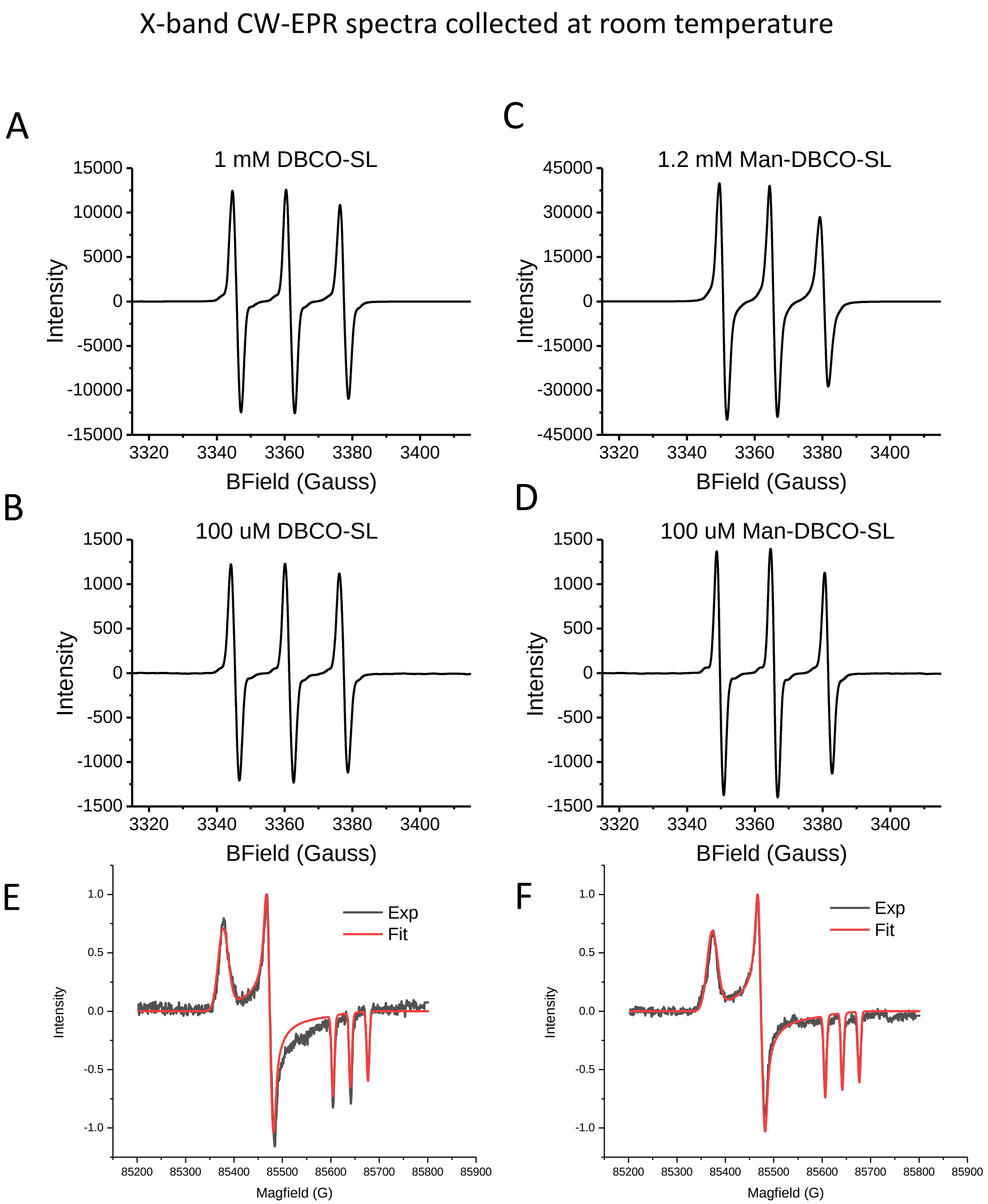

Figure SI-1. CW EPR spectra for DBCO-SL **3** and **3-1** conjugate at room temperature at 9.5 GHz and frozen at 100K at 240 GHz. 100G X-band EPR spectra of **3** in DMSO at (A) 1.0 mM and (B) 100 µM concentrations; and **3-1** conjugate in DMSO in 1:2:1 DMSO:ethanol:PBS buffer at (C) 1.2 mM and (D) 100 µM concentrations. 240 GHz EPR spectra of (E) **3** and (F) **3-1** conjugate at 1.0 and 1.2 mM, respectively, with simulations shown in red.

- **Control EPR spectra**

For each batch of cells treated with **1** or **2**, cells were split prior to feeding to generate a control sample to which **3** was allowed to react under analogous conditions to SL of treated cells. Control spectra obtained from replicate batches are shown below. There were cases where little to no

background labeling occurred and those results are also included as there were cases when no background labeling occurred when azido sugars were modified. The appropriate control spectrum was substracted from the EPR spectrum obtained for the treated cells.

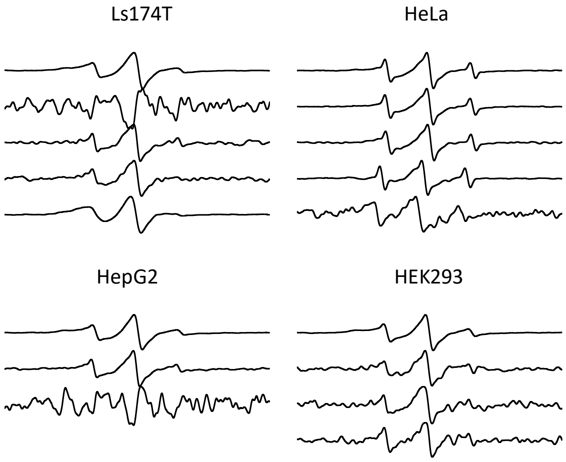

Figure SI-2. Stack plots of 100G X-band EPR spectra for DBCO-SL (**3**) only and various cells without treatment that served both as controls for subtracting from treated spectra and an indication of when spin-label solutions needed to be freshly remade. Spectra within a stack are representative of a separate batch of cells grown and treated with DBCO-SL (**3**). Initially, spin-label was dissolved in DMSO and stored in the freezer. After noting that spin-label effectiveness degraded overtime, our procedures were modified to keep the SL in solid powdered form frozen with batches solubilized in DMSO freshly for each labeling reaction. We note that the background labeling of various cells differed somewhat among cells but was consistent for a given cell line.

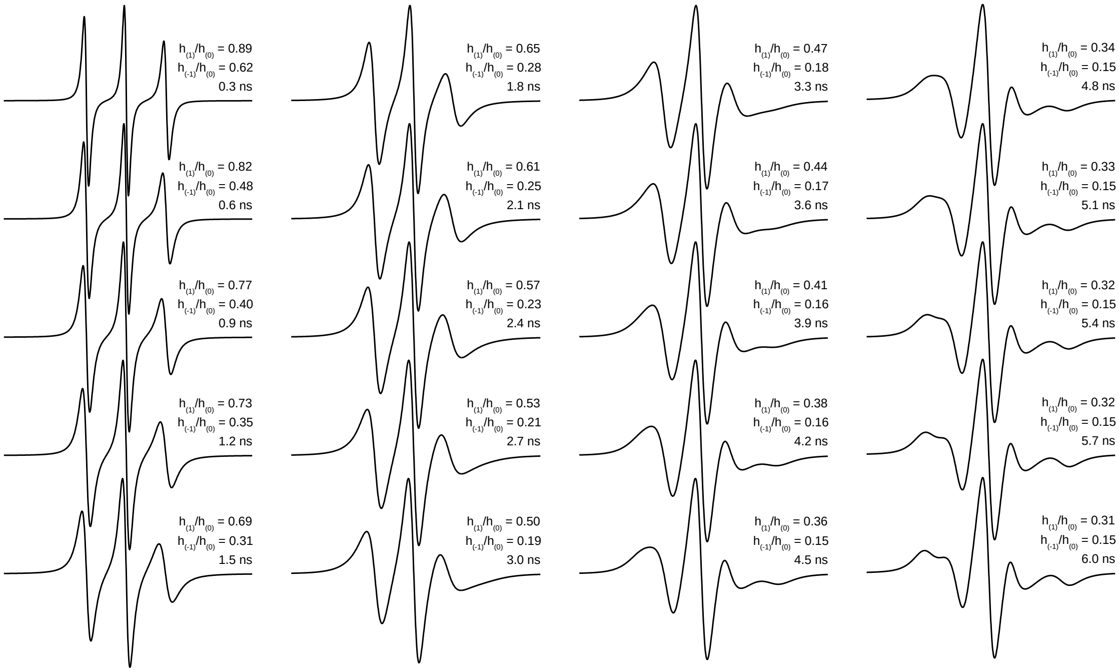

Figure SI-3. Library of simulated spectra showing how X-band line shapes change as a function of correlation time. Tensor components used for the simulation are those given in Table SI-1 with a 2G line broadening and S=0.

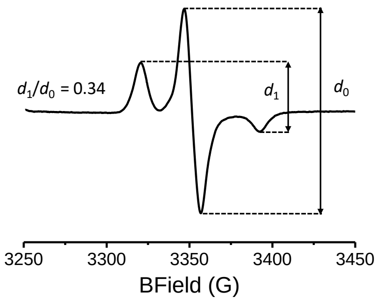

Figure SI-4. 200 G Low T (100K) spectrum of DBCO-SL/**1** (200 µM)-treated Ls174T cells showing the lack of dipolar interactions because the d_1/d_0 ratio of 0.34 indicates distances > 20-24 Ang. (*Biochemistry* 1999, 38, 32, 10324–10335).

II. EPR spectra for dose dependent experiments

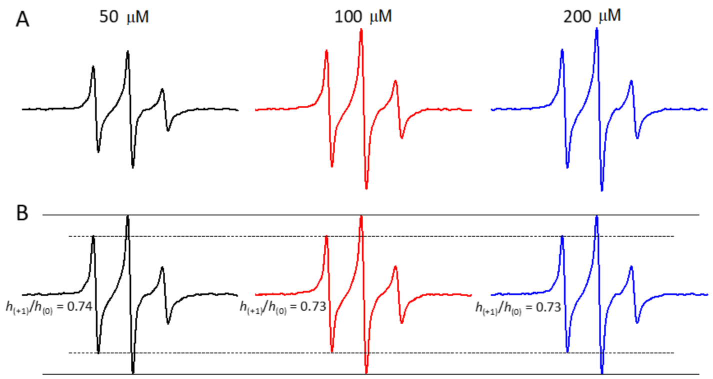

Figure SI-5. (A) 100G X-Band control-subtracted, area-normalized EPR spectra of HepG2 cell treated with 50, 100, and 200 µM of $Ac_4ManNAz$ (**1**) and then with 100 µM of DBCO-SL (**3**), showing $Ac_4ManNAz$-dependent increases in the total spin count. (B) EPR spectra of the treated HepG2 cell plotted with normalized central line intensity (solid lines) and mobility parameters showing that although there is an increase in spin-label incorporation as more sugar is added to the growth media, the mobility of the nitroxide SL does not change in a systematic way within error.

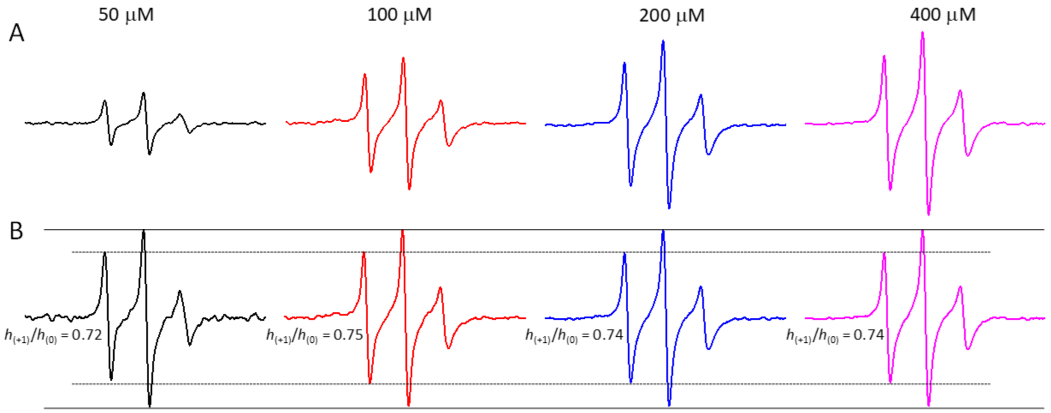

Figure SI-6. (A) 100G X-Band control-subtracted, area-normalized EPR spectra of HeLa cell treated with 50, 100, and 200 µM of $Ac_4ManNAz$ (**1**) and then with 100 µM of DBCO-SL (**3**), showing $Ac_4ManNAz$-dependent increases in the total spin count. (B) EPR spectra of the treated HeLa cell plotted with normalized central line intensity (solid lines) and mobility parameters showing that although there is an increase in spin-label incorporation as more sugar is added to the growth media, the mobility of the nitroxide SL does not change in a systematic way within error.

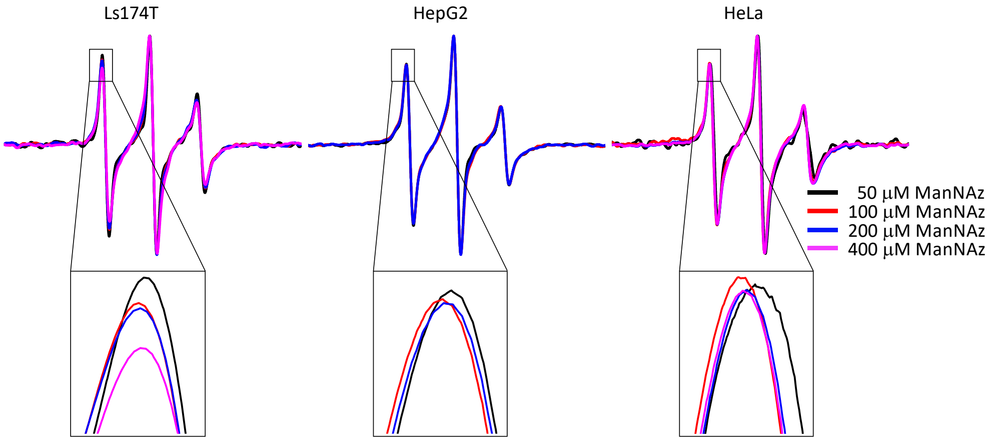

Figure SI-7. Overlays of control-subtracted and central intensity-normalized EPR spectra of Ls174T, HepG2, and HeLa cells, respectively, treated with various concentrations of Ac_4ManNAz and then with 100 μM of DBCO-SL (**3**), indicating that only Ls174T cell showed concentration-dependent systematic line shape changes of the $h_{(+1)}$ transition.

III. EPR spectra for labeled cells after enzyme treatment

After LS174T cells were incubated with **1** for 48 h, they were treated with DBCO-SL (**3**) using the method described earlier. The spin modified cells were then centrifuged at 300 x g for 5 mins, and the cell pellet was collected and resuspended in a final volume of 200 μL of DMEM media (no FBS) containing 100 U (2 μl) of α2-3,6,8,9 Sialidase A and 20 μl of 10X Glycobuffer 1® (NEB) per manufacturer's protocol. This cell suspension was incubated at 37 ºC for 1 h with continuous shaking at 120 rpm. Immediately after that, the cell suspension was centrifuged at 300 x g for 5 mins and the supernatant was carefully withdrawn and analyzed on an EPR instrument. Thereafter, the cell pellet was washed with FACS buffer three times, and finally, the resultant cell sample was subjected to EPR analysis as reported earlier. The same experimental procedure was followed for PNGaseF treatment of compound **1** modified and spin-labeled cells except for the different enzyme concentration used, which was 1000 U (2 μl) and 20 μl of 10X Glycobuffer 2® (NEB).

Sialidase and PNGaseF treatments of **1**/DBCO-SL-treated Ls174T cells resulted in nearly (~90%) reduction of the of the EPR signal intensity arising from the cells, with remaining SL-signals from cells having very low intensity (Figures SI-8B and 8C). Moreover, the resultant supernatants showed significant EPR signals (spectra shown in Figures SI-8D and 8E), resulting from the SL-labeled sialic acid and *N*-glycans released from the cells. Their line shapes are reflective of highly isotropic motion for labeled sialic acids ($h_{(+1)}/h_{(0)} = 1.05$) and with slightly restricted mobility for *N*-linked glycans ($h_{(+1)}/h_{(0)} = 0.88$; $h_{(-1)}/h_{(0)} = 0.72$).

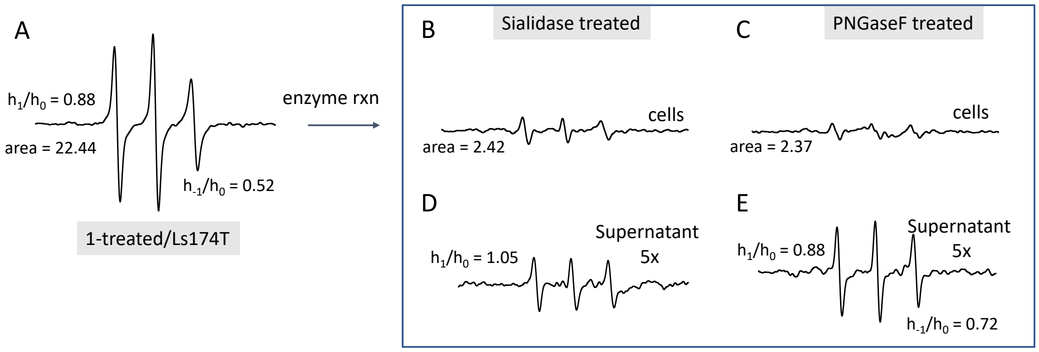

Figure SI-8. 100 G X-band EPR spectra for **1**/DBCOSL-treated Ls174T cells revealing nearly complete (~90%) removal of the EPR signal by incubation with sialidase or PNGaseF, which remove terminal sialic acid moieties and N-linked glycans, respectively. Spectra obtained for fixed cells labeled with **3** (A) and then reacted with sialidase (B) or PNGase (C) and the collected supernatant spectra shown in D and E, respectively (5x y-axis scaling due to dilution). Interpretations are discussed within the main text.

IV. EPR Spectral Simulation Results (Room temperature X-band data of cells)

Room temperature X-band EPR spectra were simulated using *chili* and *esfit* functions of EasySpin software (S. Stoll and A. Schweiger, *J. Magn. Reson.* 2006, *178*, 42-55). Spectra were originally simulated without low-temperature, high-field spectra for determining *g*- and *A*-tensor values for the new SL, and initial parameters were obtained by simulations of control-subtracted **3**/**1**-treated Ls174T spectra. Then these initial values for *g*- and *A*-tensor were utilized in a global fitting of all spectra such that all spectra have a single set of *g*- and *A*-tensor with reasonable correlation times. The best fit of *A*- and *g*-tensor values were: $g_{xx} = 2.0070$, $g_{yy} = 2.0062$, $g_{zz} = 2.0033$, $A_{xx} = 6.7$ G, $A_{yy} = 6.7$ G, and $A_{zz} = 35$ G. Upon collection of high-field data (delayed because of COVID-19 shutdown), **we find these values are in excellent agreement with those listed in Table SI-1**, and give us great confidence in our simulation procedure for X-band line shapes. Other parameters used in the EPR line shape simulation include linewidth (which can be reflective of spin-spin interactions), correlation time of motion (τ_c), and the ordering potential C20, which was used to calculate the motional order parameter *S*. Simulations of the spectra were attempted first using a 1-component setup. If 1-component fit simulations could not recapitulate the features of a spectrum, then 2-component simulations were utilized, which contained a fast motion component to describe the sharp peaks and a slower motion component to capture the broadened areas of the spectrum. We incorporated an ordering potential in all simulations because we did not want to assume isotropic motion. Note, most if not all values of S approach 0, indicative of highly isotropic motion. Note, that for GlcNAc spectra, two component fits gave optimal solutions in all cases. For simulations of SL on 1-treated cells, in many cases either single component or two component spectral simulations could recapitulate the features. Hence, we show both results here and give alternative explanations.

Table SI-2. Simulation results from both 1-component fit and 2-component fits of control-subtracted spectra of Ls174T Ac_4MasnNAz and DBCO-SL-treated cells.

Parameters	1-comp fit	2-comp fit	
		Comp 1	Comp 2
FWHM (G)	2.28	2.33	0.18
τ_c (ns)	0.9	0.8	3.9
Percentage (%)	100	82	18
C20	0.02	0.01	0.16
S	0.0016	0.0008	0.013
RMSD	6.488	5.282	

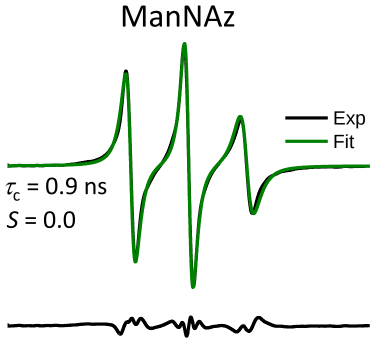

Figure SI-9. 1-component fit of the control-subtracted experimental EPR spectrum of Ac_4ManNAz/DBCO-SL (**3**)-treated Ls174T cell. The two-component fit spectrum and residual data are shown in Figure 3 of the main document.

Table SI-3. Two-component fit simulation results of the control-subtracted EPR spectrum of Ac_4GlcNAz/DBCO-SL treated Ls174T cell

Parameters	Comp 1	Comp 2
FWHM (G)	2.13	1.22
τ_c (ns)	0.87	4.1
Percentage (%)	30	70
C20	0.01	0.85
S	0.0008	0.072
RMSD	6.995	

Table SI-4. Two-component fit simulation results of the control-subtracted EPR spectrum of Ac_4GlcNAz/DBCO-SL - treated HeLa cell

Parameters	Comp 1	Comp 2
FWHM (G)	1.85	1.22
τ_c (ns)	0.88	3.8-4.1
Percentage (%)	47-49	53-51
C20	0.067	0.108
S	0.0053	0.0087
RMSD	0.903	

Table SI-5. One-component fit simulation results of the control-subtracted EPR spectra of Ls174T cells treated with 50, 100, 200, and 400 μM of $Ac_4ManNAz$, respectively, and then with 100 μM of DBCO-SL (**3**)

Parm.	Fixed τ_c				Fixed linewidth			
	50 μM	100 μM	200 μM	400 μM	50 μM	100 μM	200 μM	400 μM
FWHM (G)	2.00	2.43	2.45	2.71	2.28	2.28	2.28	2.28
τ_c (ns)	0.90	0.90	0.90	0.90	0.77	1.00	1.01	1.18
S	0.00016	8.00E-5	0.0003	0.006	0.00016	6.00E-5	0.00016	8.00E-7
RMSD	0.078	0.075	0.072	0.075	0.078	0.075	0.072	0.074

Table SI-6. One-component fit simulation results of the control-subtracted EPR spectra of HepG2 cells treated with 50, 100, and 200 μM of $Ac_4ManNAz$, respectively, and then with 100 μM of DBCO-SL (**3**)

Parm.	Fixed τ_c			Fixed linewidth		
	50 μM	100 μM	200 μM	50 μM	100 μM	200 μM
FWHM (G)	1.91	1.99	2.01	2.28	2.28	2.28
τ_c (ns)	0.90	0.90	0.90	0.80	0.83	0.85
S	5.00E-7	1.10E-6	2.55E-6	-1.11E-7	4.01E-7	5.56E-7
RMSD	0.048	0.025	0.041	0.049	0.026	0.042

Table SI-7. One-component fit simulation results of the control-subtracted EPR spectra of HeLa cells treated with 50, 100, 200, and 400 μM of $Ac_4ManNAz$, respectively, and then with 100 μM of DBCO-SL (**3**)

Parm.	Fixed τ_c				Fixed linewidth			
	50 μM	100 μM	200 μM	400 μM	50 μM	100 μM	200 μM	400 μM
FWHM (G)	2.12	2.09	2.18	2.17	2.28	2.28	2.28	2.28
τ_c (ns)	0.90	0.90	0.90	0.90	0.96	0.94	0.91	0.96
S	0.100	0.009	0.004	3.57E-8	-1.30E-7	-4.81E-8	-1.30E-7	1.50E-7
RMSD	0.035	0.024	0.023	0.023	0.066	0.036	0.042	0.045

Table SI-8. Two-component fit simulation results of the control-subtracted EPR spectra of Ls174T cells treated with 50, 100, 200, and 400 μM of $Ac_4ManNAz$, respectively, and then with 100 μM of DBCO-SL (**3**)

Parm.	50 μM		100 μM		200 μM		400 μM	
	Comp 1	Comp 2	Comp 1	Comp 2	Comp 1	Comp 2	Comp 1	Comp 2
FWHM (G)	1.01	14.0	1.28	-1.65	1.31	-1.67	1.41	-1.17
τ_c (ns)	0.9	4.0	0.9	4.0	0.9	4.0	0.9	4.0
Percentage (%)	~100	~0	91	9	89	11	80	20
S	7.65E-6	0.01	5.34E-5	0.01	2.3E-4	0.007	1.19E-7	0.01
RMSD	0.059		0.055		0.052		0.054	

V. ^{1}H-NMR and MS spectra of compound 3

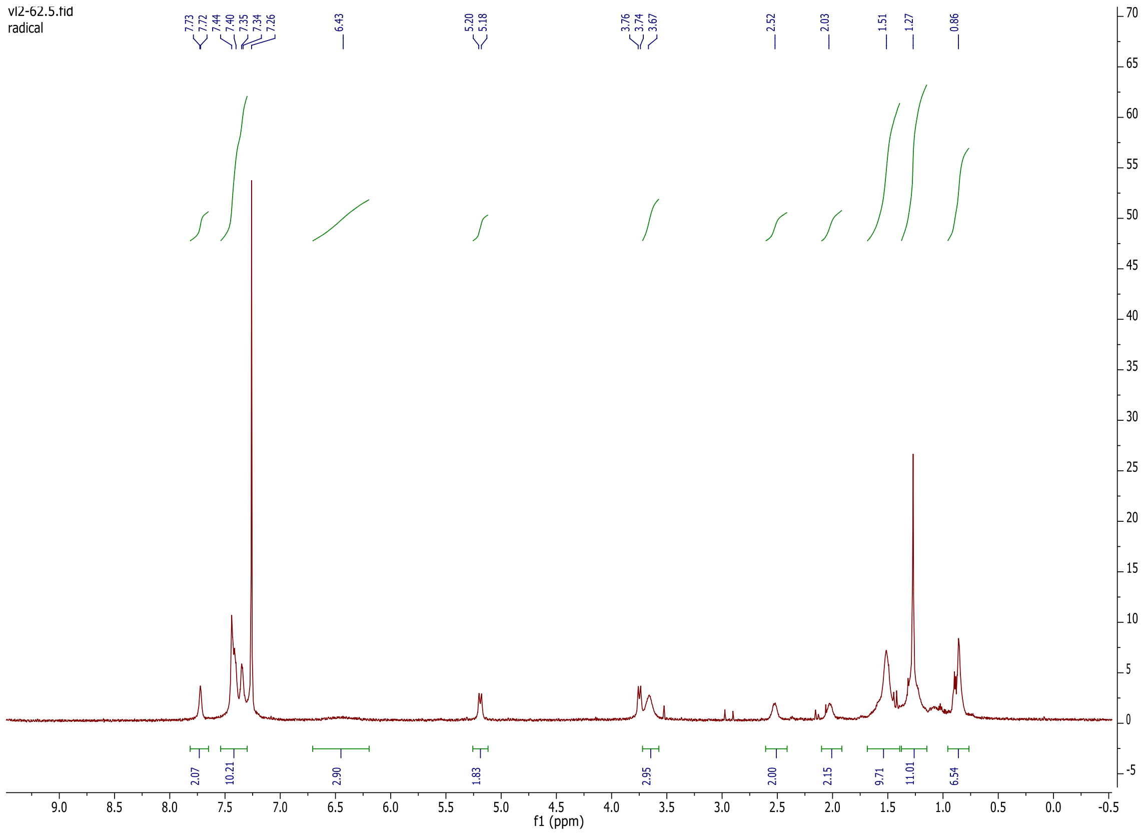

Figure SI-10. ^{1}H NMR (600 MHz, $CDCl_3$) of compound **3**.

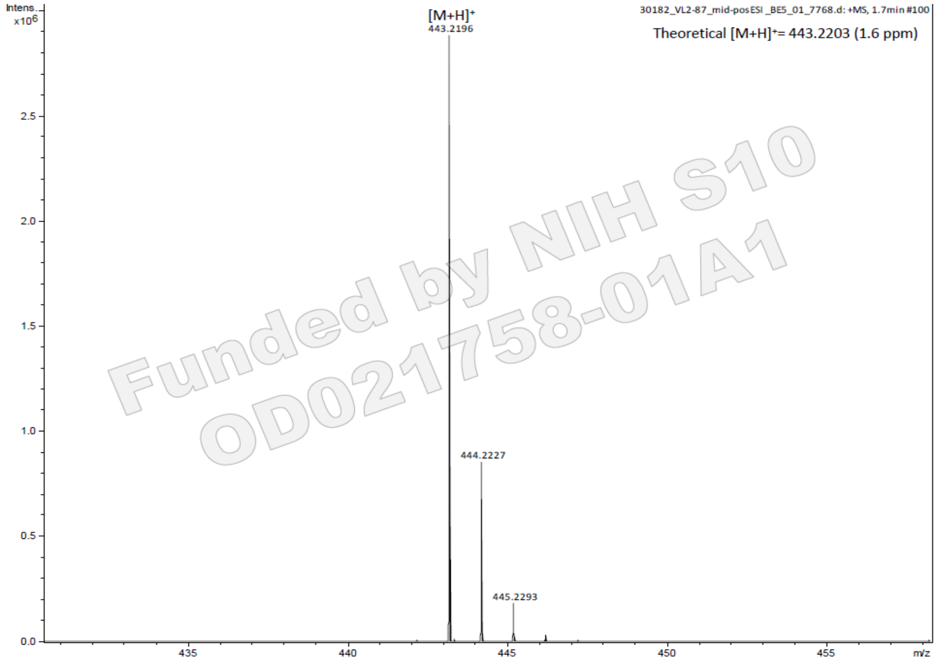

Funded by NIH S10 OD021758-01A1

Figure SI-11. HR ESI-MS of compound **3**.
